# Evolution of Fungal U3 snoRNAs: Structural Variation and Introns

**DOI:** 10.3390/ncrna3010003

**Published:** 2017-01-05

**Authors:** Sebastian Canzler, Peter F. Stadler, Jana Hertel

**Affiliations:** 1 Bioinformatics Group, Department Computer Science, and Interdisciplinary Center for Bioinformatics, University Leipzig, Härtelstrasse 16-18, D-04107 Leipzig, Germany; sebastian@bioinf.uni-leipzig.de; 2 German Centre for Integrative Biodiversity Research (iDiv) Halle-Jena-Leipzig, Competence Center for Scalable Data Services and Solutions, and Leipzig Research Center for Civilization Diseases, University Leipzig, D-04107 Leipzig, Germany; 3 Max Planck Institute for Mathematics in the Sciences, Inselstraße 22, D-04103 Leipzig, Germany; 4 Fraunhofer Institute for Cell Therapy and Immunology, Perlickstrasse 1, D-04103 Leipzig, Germany; 5 Department of Theoretical Chemistry of the University of Vienna, Währingerstrasse 17, A-1090 Vienna, Austria; 6 Center for RNA in Technology and Health, University of Copenhagen, Grønnegårdsvej 3, 1870 Frederiksberg C, Denmark; 7 Santa Fe Institute, 1399 Hyde Park Road, Santa Fe, NM 87501, USA; 8 Helmholtz Centre for Environmental Research—UFZ, Young Investigators Group Bioinformatics and Transcriptomics Permoserstraße 15, D-04318 Leipzig, Germany; jana.hertel@ufz.de

**Keywords:** small nucleolar RNA, pre-rRNA processing, RNA secondary structure, spliceosomal introns, RNA–RNA interactions, evolution

## Abstract

The U3 small nucleolar RNA (snoRNA) is an essential player in the initial steps of ribosomal RNA biogenesis which is ubiquitously present in Eukarya. It is exceptional among the small nucleolar RNAs in its size, the presence of multiple conserved sequence boxes, a highly conserved secondary structure core, its biogenesis as an independent gene transcribed by polymerase III, and its involvement in pre-rRNA cleavage rather than chemical modification. Fungal U3 snoRNAs share many features with their sisters from other eukaryotic kingdoms but differ from them in particular in their 5’ regions, which in fungi has a distinctive consensus structure and often harbours introns. Here we report on a comprehensive homology search and detailed analysis of the evolution of sequence and secondary structure features covering the entire kingdom Fungi.

## 1. Introduction

The U3 small nucleolar RNA (snoRNA) is a box C/D snoRNA with an exceptional structure. Like many other snoRNAs it is involved in pre-rRNA processing. However, in contrast to typical box C/D snoRNAs [1,2] it does not guide 2${}^{\prime }$O-methylation. Instead it acts as a an RNA-chaperone mediating structural changes to ensure the correct pre-rRNA cleavage. A comprehensive survey showed that it is ubiquitously present in Eukaryotes [3]. Although its sequence is overall only loosely conserved, it harbours eight highly conserved box motifs including the box C, C${}^{\prime }$, D, and D${}^{\prime }$ motifs characteristic for box C/D snoRNAs. The molecule also exhibits a strongly constrained secondary structure that nevertheless shows lineage-specificity of the canonical 10 stems as well as frequent extensive expansions [3]. U3 snoRNAs share important features with small nuclear RNAs (snRNAs). In particular, they are independently transcribed using a promoter comprising a TATA box and a Homol D box [4]. In addition they feature a 2,2,7-trimethylguanosine cap [5].

**Figure 1:**
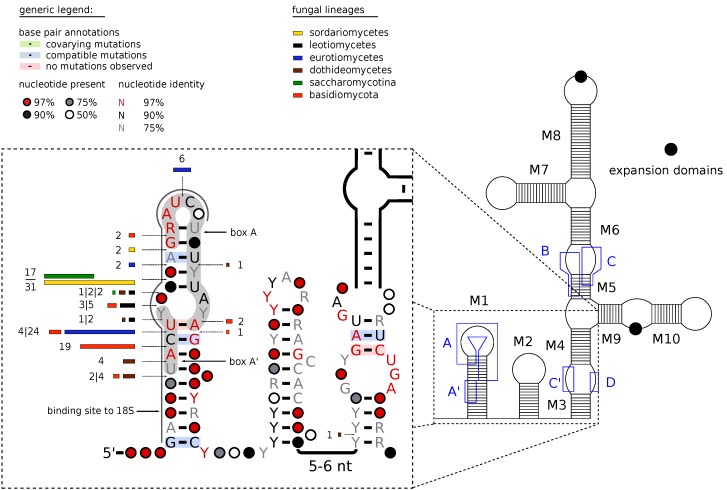
Overview map of U3 snoRNAs. The schematic overview on the left (redrawn from Marz and Stadler [3]) defines the nomenclature of the 10 stem and hairpin regions, and indicates the location of the conserved box motifs. The detail on the right, summarizes the particular features of fungal U3 snoRNAs, including the more complex structure of M1. It also indicates the insertion points of introns, which are predominantly located within the 5’ arm of the stem-loop structure M1. The lengths of the bars and the corresponding numbers give the number of species with introns at a particular location, the color indicates their membership in one of the major clade. The drawing was constructed using R2R [14].

Fungal U3 snoRNAs deviate from the eukaryote consensus in particular close to their 5${}^{\prime }$ end, see Figure 1 below. The region consisting of two leading hairpins interacts with the rRNA precursor [6,7,8]. Specifically, the 5${}^{\prime }$ half of hairpin 1 (including box A${}^{\prime }$ and in part box A) forms base pairs with a region at the 5${}^{\prime }$ end of the later mature 18S rRNA (U9 to C25 in *Saccharomyces cerevisiae*), while the hinge between the first and the second hairpin binds to the 5${}^{\prime }$ external transcribed spacers (ETS). A special feature of fungal U3 snoRNAs is the presence of introns, first discovered in both *S. cerevisiae* U3 genes, which are excised by the canonical pre-mRNA splicing machinery [9]. Similarly, there are three intron-interrupted copies in *Neurospora crassa* [7]. In contrast, both U3 genes in fission yeast are intronless [10]. So far, the U3 snoRNA and the U6 snRNA [11,12,13] are the only small structured RNAs that are known to be often interrupted by an intron.

## 2. Methods

### 2.1. Homology Search

We surveyed a total of 147 fungal genomes from Microsporidia, Mucoromycotina, Blastocladiomycota, Basidiomycota, and Ascomycota; a complete list is supplied in the Supplemental Material (http://www.bioinf.uni-leipzig.de/publications/supplements/16-018). The 54 fungal U3 snoRNAs from 52 distinct organisms reported in [3] were used as initial query set. These were aligned with muscle [15] and subsequently used to construct a covariance model (CM) to search the 147 fungal organisms with infernal v1.1 [16,17]. In the second step, dedicated CMs were constructed for each of the major fungal clades starting from muscle alignments comprising both previously published U3 sequences and the homologs detected in the initial infernal search. Novel sequences found by these CMs were furthermore added to the alignments.

### 2.2. Secondary Structure Prediction

Structural models were computed and iteratively improved using mLocARNA [18], RNAz [19], as well as RNAsubopt and RNAalifold from the ViennaRNA package [20] as well as some manual curation. Lineage-specific, structure annotated alignments are provided in the Supplemental Material and have been submitted to the Rfam (http://rfam.xfam.org/) database.

### 2.3. U3-Target Interactions

Base pairing between U3 and potential target sites were computed with RNAduplex a component of the ViennaRNA package [20] designed to compute RNA-RNA interactions. The experimentally validated U3 interaction region in budding yeast covers the first 30 nucleotides of the budding yeast 18S rRNAs [8]. Homologous sequences were retrieved from 85 fungal species for which the complete 18S rRNA sequences were available. To simplify the interaction prediction only the 5${}^{\prime }$terminus of the U3 snoRNA was used since RNAduplex does not handle complex internal structures of the interaction partners. As a control we used random sequences with the same length and nucleotide background distribution as the 18S rRNA fragments.

### 2.4. Characterization of Sequence Motifs

Introns were identified using meme [21] to retrieve motifs of length 7 nt (5${}^{\prime }$splice site and branch site) and 5 nt (3${}^{\prime }$splice site), respectively. A survey of 11.000 introns from five fungal species [22] reports typical properties of fungal introns such as length distributions and sequence patterns of splice and branch sites. These patterns are used for comparison with the predicted U3 intron sequences.

## 3. Results

U3 snoRNA genes were identified in all 147 genomes included in this study, supporting the indispensable nature of U3 in rRNA biogenesis. We found a total of 310 genes, i.e., the U3 snoRNAs, similar to snRNAs, tend to appear in multiple genomic copies. Almost all investigated genomes contain three or fewer copies. Notable exceptions are *Schizosaccharomyces japonicus* and *Allomyces macrogynus* with 11 and 10, resp., nearly identical paralogs. Duplications are evolutionarily recent and appear to be species- or genus-specific. There is no evidence for diverged families of paralogs. Instead, paralogous copies are presumably subject to concerted evolution [23] similar to other ncRNA families including tRNAs.

### 3.1. Sequence and Structure Motifs

**The secondary structures** of U3 snoRNAs vary substantially between kingdoms [3]. Even within Fungi there are extensive lineage-specific peculiarities such as additional stem-loop elements, missing helices, and large unstructured insertions, see Table 1 for a detailed summary. All fungal sequences share the distinctive two-hairpin motif (marked as M1 and M2 in Figure 1) at the 5${}^{\prime }$ end and the following two stems M3 and M4. Microsporidia share a stem-loop structure including helix M5 and a loop region containing the C box motif. In contrast, helix M5 is missing in Hypocreomycetidae and Saccharomycotina. These feature instead a large multi-branch loop harbouring the box B and C motifs. The following structures including stems M6 through M10 are subjected to major lineage and even species-specific changes. Several lineages evolved M6 (Blastocladiomycota, Dothideomycetes) or M9 (Blastocladiomycota, Taprhinomycotina) into a single hairpin. In cases where either one or both of these hairpins are disrupted by an interior loop with at least two unpaired nucleotides on both sides, the original helix is split into a double-helix-hairpin combination (M6/M8 and M9/M10). This variation concerns in particular the majority of the Eurotiomycetes. In Saccharomycotina, Leotiomycetes, and Sordariomycetes, an additional hairpin M7 emanates from the interior loop between helices M6 and M8. The basidiomycete *Rhodotorula minuta* has a 50 nt insert in the loop of stem M6, the yeast *Kluyveromyces lactis* shows an even larger 80 nt insert in M8, while another yeast, *Candida glabrata* has an extra stem of about 50 nt between M9 and M10.

**The characteristic sequence motifs** offer no surprises in the fungal kingdom. Motif A${}^{\prime }$ = TACTY is nearly perfectly conserved, while A = GYATCW is more variable. Both C and C${}^{\prime }$ largely adhere to the consensus RTGATGA typical for box C/D snoRNAs. As in other box C/D snoRNAs, box C${}^{\prime }$ allows more variability and is better represented by the more relaxed consensus WYGATGA. The pattern for box B is quite variable at both ends but conserves the core motif AGYGA. Box D = CTGA is very stringently conserved and matches the pattern perfectly in almost all of the 310 sequences. Not unexpectedly, box D${}^{\prime }$ has the same consensus with higher levels of variation. Appendix A summarizes the box motifs in graphical form.

**Figure 2:**
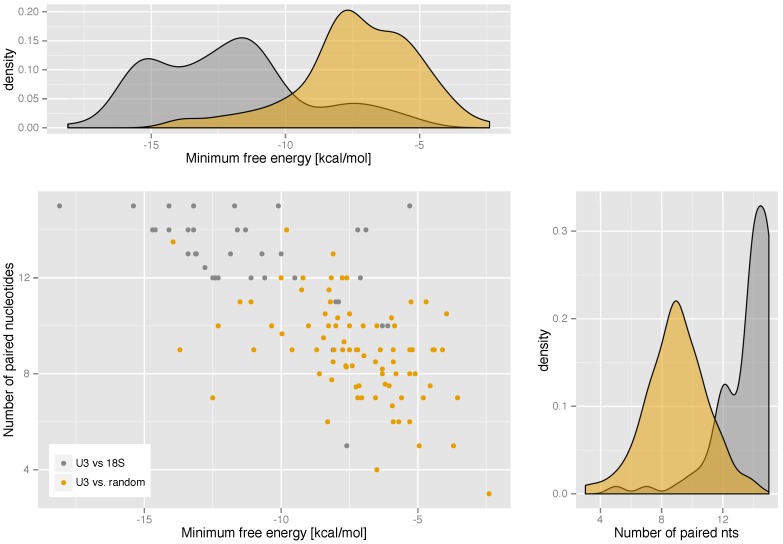
U3:pre-rRNA interactions are significantly more stable than random RNA:RNA interactions with the same sequence composition. Energies and minimum free energy interaction structures are computed with RNAduplex. The scatter plot shows the expected correlation between interaction energies and number of base pairs.

**The guiding potential** of the predicted U3 snoRNA genes can be quantified by the number of base pairs and the interaction energy between the 5${}^{\prime }$ region of the U3 snoRNA and the 5${}^{\prime }$ end of the 18S rRNA. A comparison with a random control shows that the interaction is consistently more stable ($\bar{\Delta G}=-12.17$ kcal/mol) and comprises more base pairs ($\bar{\ell }=13.5$) compared to the randomized control ($\bar{\Delta G}=-7.30$ kcal/mol and $\bar{\ell }=8.9$). In fact, Figure 2 shows that the distribution observed for the U3:pre-rRNA interactions is well separated from the randomized background.

### 3.2. Introns within U3 snoRNA Genes

Of the 310 genes from 147 organisms 138 from 78 genomes are interrupted by at least one intron. Intron-bearing genes are not found in early branching lineages such as Microsporidia, Mucoromycotina, Chytridiomycota, or Blastocladiomycota. They are broadly distributed across the Basidiomycota but not present in Taphrinomycotina. In Pezizomycotina they are found in the vast majority of analyzed organisms. Among the Saccharomycotina, only Saccharomycetaceae have introns. U3 genes both with and without introns are present in the *Kluyveromyces* genus [24]. A more detailed summary is included in Table 1. Four genes in three species appear to contain two introns per transcript (*Baudoinia compniacensis*, *Cucurbitaria berberidis*, and both U3 genes in *Rhodotorula graminis*). In eight genomes we observed multiple copies of the U3 gene of which at least one is intronless and another one contains an intron, e.g., *Coprinopsis cinerea*, a basidiomycete, carries two U3 transcripts where one is intron interrupted. The remaining seven species are spread over Dothideomycetes (*Alternaria brassicicola*, 2/1), Leotiomycetes (*Sclerotinia sclerotiorum*, 2/1; *Botrytis cinerea*, 2/1), and Sordariomycetes (*Fusarium oxysporum*, 3/1; *Fusarium verticillioides*, 3/1; *Nectria haematococca*, 3/2; *Acremonium alcalophilum*, 2/1).

The U3 introns have an average length of 92 nt, approximately matching the typical intron length in most fungi [22]. Almost all introns (136, i.e., 98.54%) have canonical 5${}^{\prime }$GT-AG3${}^{\prime }$ junctions. The two exceptions are 5${}^{\prime }$GC-AG3${}^{\prime }$ in *Pyrenophora tritici-repentis* and 5${}^{\prime }$GC-GG3${}^{\prime }$ in *Nadsonia fulvescens*. Notably, GC-AG junctions have been observed a common exception also in large surveys of fungal introns [22,25]. Nearly two thirds (88/136) of the canonical donors share the consensus pattern GTRWGT, where the underlined nucleotides are absolutely conserved. Almost all of the remaining donor have a mismatch in exactly one of the other three positions. All except one acceptor sites share YAG with a strong preference of CAG over TAG (129 versus 8 introns). The patterns conform very well to the consensus motifs derived from large sets of introns [22,26]. The branch site within the intron provides an adenosine to perform the first nucleophilic attack at the donor site and is essential for lariat formation. Its consensus sequence motif in fungi is RCTRAY [22]. The predicted U3 introns exhibit a matching consensus pattern RCTRAC in 129/138 cases. The distance between branch and acceptor sites is strongly constrained, varying between 6 to 36 nt in fungal intron [22,27]. The average distance for the U3 introns is 15 nt and only 4% have a branch point to acceptor distance exceeding 36 nt. More detailed information including splice site positions, splice site and branch site motifs can be found in the supplement material. The U3 consensus splice site and branch points motifs are shown in Appendix A. In summary, the computational analysis of U3 introns consistently shows that they are normal spliceosomal introns although direct experimental evidence so far is available only for *S. cerevisiae* and *N. crassa* [7,9].

The introns are inserted almost exclusively in the most-5${}^{\prime }$ stem-loop structure M1, which also harbours the essential box A and A${}^{\prime }$ motifs ( Figure 1). 133 of 138 introns are placed in the 5${}^{\prime }$ half. Three distinct positions are characteristic for nearly all introns found in Basidiomycota, Eurotiomycetes, and Saccharomycetaceae/Sordariomycetes. All other positions are rarely used. Three of the four 2nd introns are located on the 3${}^{\prime }$ side of the stem-loop M1. The only exception is the 2nd intron in *Baudoinia compniacensis*, which is inserted in stem M3 upstream of box C${}^{\prime }$.

The tight clustering of intron locations in the U3 snoRNA sequence suggests that at least large groups of the introns are homologous, dating back to the same insertion event. Due to the rapid rate of sequence evolution in introns direct evidence for a common origin can be obtained from sequence similarity however only for closely related species. Introns with the *Penicillium* or *Saccharomyces* genera, for example exceed 75%. Between *Aspergillus* and *Penicillium* we still observe between 40% and 60%. On the other hand there is also evidence for intron sliding [28]. The intron sequences of Dothideomycetes show high levels of sequence similarity but vary slightly in their insertion position. These variations do not seem to be correlated with the relationships among the Dothideomycetes but appear essentially random. Introns in paralogs almost always have very similar sequences pointing at recent, lineage-specific duplication events at the DNA level. In the four species with two introns there is no recognizable homology between the 1st and the 2nd intron. A detailed heatmap showing pairwise intron sequence similarities is given in the supplement material. The congruence of intron similarities with major phylogenetic groups is also consistent with only a few, early intron gain events.

## 4. Discussion

The U3 snoRNA is much better conserved in fungi and in eukaryotes in general than all other box C/D snoRNAs. Despite the presence of several important, well-conserved sequence motifs and well conserved core secondary structure elements, it shows extensive variability and clade-specific peculiarities. Among its well-preserved key features is also the propensity of its 5${}^{\prime }$-terminus to interact with the pre-18S rRNA. The observed flexibility of the U3 snoRNA sequence is consistent with the high throughput screening of fitness effects random mutations within the U3 snoRNA gene of about 60,000 *S. cerevisiae* strains, which found that typically effects of individual mutations are small but may have very large epistatic effects acting in particular to maintain base pairing patterns [29].

Fungal U3 genes are the only snoRNAs that are frequently interrupted by introns. These share canonical pre-mRNA splice sites and are clearly processed by the spliceosomal machinery. Introns have been inserted almost exclusively in the 5${}^{\prime }$-side of the first stem-loop structure, which also harbours the site of interaction with the 18S rRNA precursor. It is unlikely that the intron has an impact on the role of U3 in rRNA maturation since closely related species and even paralogs from the species may differ in the presence of the intron.

U3 introns appear only in the crown group fungal lineages. Introns may have been inserted only a single time in fungal history [7]. If this was the case, they have been lost independently in many lineages. The variation in the intron position then has to be explained as the result of intron sliding. Intron sliding, however, is rare in yeasts [30]. It is unlikely, furthermore, that sliding could explain the distant insertion sites on the 3${}^{\prime }$ side of M1. Alternatively, there may have been independent insertion events. These two scenarios cannot be distinguished based on the available data because intron sequences have diverged beyond detectable similarities between major clades.

Although the 5${}^{\prime }$ part of the U3 gene, including helices M1–M3, is fairly well-conserved among the fungi, their 3${}^{\prime }$ end is subject to major lineage-specific variations. Furthermore, several species developed large unstructured or hairpin-like inserts indicating an ongoing expansion of the transcript size. However, neither these significant structural diversities nor the recurrent insertion of intronic sequences seem to impede or decrease the base pairing potential of U3 in with the rRNA precursor.

## Figures and Tables

**Table 1:** Variation of U3 snoRNA secondary structures across the major fungal lineages.

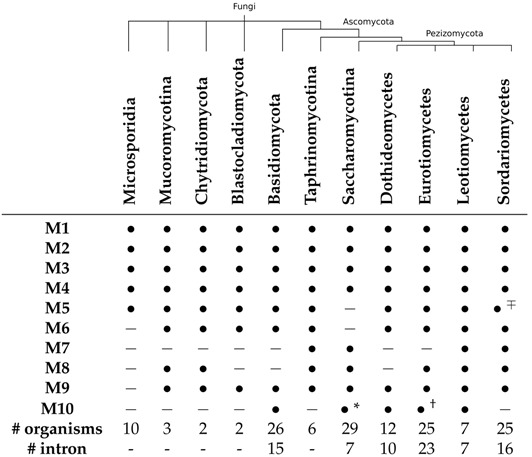

The nomenclature of hairpins and stem-loop follows Marz and Stadler [3], see the r.h.s. panel in Figure 1. Variations in subclades are indicated as follows: * not present in Saccharomycetaceae; ${}^{†}$ not present in *Penicillium*; ${}^{\mp }$ not present in Hypocreomycetidae.

**Figure A1:**
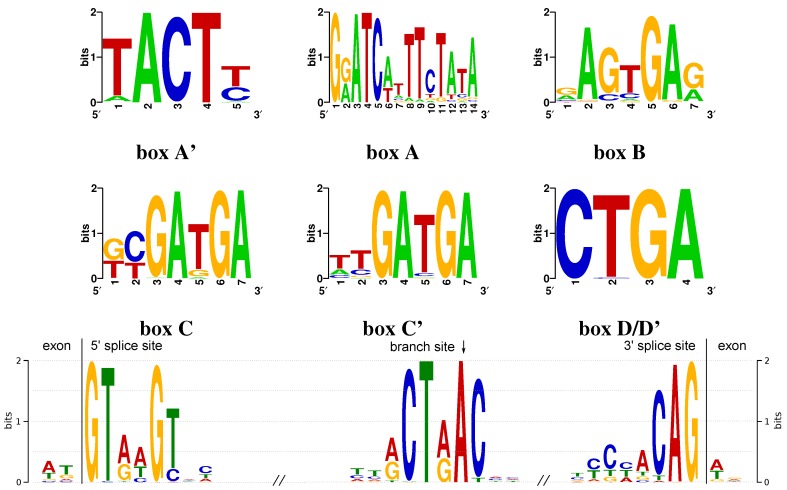
Sequence logos of conserved sequence elements in U3 snoRNAs (**above**) and in introns located in U3 snoRNA genes (**below**). All motifs were inferred with meme from all available U3 RNAs and introns, resp. The exact position of the splice junctions are indicated.
